# Arsenic, Zinc, and Aluminium Removal from Gold Mine Wastewater Effluents and Accumulation by Submerged Aquatic Plants (*Cabomba piauhyensis*, *Egeria densa*, and *Hydrilla verticillata*) 

**DOI:** 10.1155/2013/890803

**Published:** 2013-09-12

**Authors:** Ahmad Farid Abu Bakar, Ismail Yusoff, Ng Tham Fatt, Faridah Othman, Muhammad Aqeel Ashraf

**Affiliations:** ^1^Geology Department, Faculty of Science, University Malaya, 50603 Kuala Lumpur, Malaysia; ^2^Civil Engineering Department, Faculty of Engineering, University Malaya, 50603 Kuala Lumpur, Malaysia

## Abstract

The potential of three submerged aquatic plant species (*Cabomba piauhyensis, Egeria densa*, and *Hydrilla verticillata*) to be used for As, Al, and Zn phytoremediation was tested. The plants were exposed for 14 days under hydroponic conditions to mine waste water effluents in order to assess the suitability of the aquatic plants to remediate elevated multi-metals concentrations in mine waste water. The results show that the *E. densa* and *H. verticillata* are able to accumulate high amount of arsenic (95.2%) and zinc (93.7%) and resulted in a decrease of arsenic and zinc in the ambient water. On the other hand, *C. piauhyensis* shows remarkable aluminium accumulation in plant biomass (83.8%) compared to the other tested plants. The ability of these plants to accumulate the studied metals and survive throughout the experiment demonstrates the potential of these plants to remediate metal enriched water especially for mine drainage effluent. Among the three tested aquatic plants, *H. verticillata* was found to be the most applicable (84.5%) and suitable plant species to phytoremediate elevated metals and metalloid in mine related waste water.

## 1. Introduction

Mining effluent waste water often has elevated concentration of metal and metalloid. This is due to the mine drainage discharge from the metal processing plant and the flushing of weathered material [1] through runoff mechanism [2]. These toxic metals can be transported into the pond, ditch, and river water and may eventually affect the lives of local people that using this water as primary source for their daily requirements [3]. Various remediation strategies may be used to treat the contaminated water, for example, through ion exchange, reverse osmosis, electrolysis, precipitation, and adsorption. However, these methods are usually high cost [4], time consuming, and labor exhaustive and may contribute to the other environmental problem (e.g., dumping site for metal waste). 

The plant based remediation technology is an emerging concept aims to accumulate and translocate metal in plant cells and thus removing metal content in water through several phytoremediation processes, namely, phytoextraction, phytodegradation, rhizofiltration, phytostabilization, and phytovolatilization [5, 6]. This technology uses various types of plants to degrade, extract, contain, or immobilize contaminants in soil [7] and water [8]. In recent years, phytoremediation techniques have received increasing attention due to cost-effective, nonintrusive, and safe alternative compared to conventional cleanup techniques [9]. 

The aquatic plant can be either floating on the water surface or submerged into the water. The floating aquatic plants accumulate contaminants by its roots [10], while the submerged plants accumulate metals by their whole body. Indirectly, the aquatic plants also may act as traps for particulate material, by slowing the water current and favoring sedimentation of suspended particles and thus influencing metal retention in water bodies [11, 12]. The ability of plants to hyperaccumulate metals and metalloids in plant mass has led to the ideas that sustainable and ecofriendly remediation strategies could be developed based on this characteristic [3]. By definition, a hyperaccumulator must accumulate at least 100 mg g^−1^ (0.01% dry wt.), Cd, As, and some other trace metals, 1000 mg g^−1^ (0.1 dry wt.) Co, Cu, Cr, Ni, and Pb and 10,000 mg g^−1^ (1% dw) Mn and Ni [13–15].

Several submerged aquatic macrophytes have been investigated for the remediation of natural and wastewater contaminated. For example, the submerged aquatic plant *Myriophyllum spicatum L*. has been reported as an efficient plant species for the metal-contaminated industrial wastewater treatment [17]. The aquatic plants *Rorippa nasturtium-aquaticum* (L.) and *Mentha* spp. accumulate arsenic from contaminated freshwater [18]. Five common aquatic plant species (*Typha latifolia, Myriophyllum exalbescens, Potamogeton epihydrus, Sparganium angustifolium,* and *Sparganium multipedunculatum*) were tested for Al phytoremediation [19]. Kamal et al. (2004) have reported an effective removal of Fe, Zn, Cu, and Hg from contaminated water using Parrot feather (*Myriophyllum aquaticum*), creeping primrose (*Ludwigia palustris*), and water mint (*Mentha aquatic*) [20]. These encouraging results regarding phytoremediation using aquatic plants gained much attention of researchers and scientists to continue exploring in this field.

The objectives of this study were (a) to assess the ability of three submerged aquatic plants (*Cabomba piauhyensis *Gardner,* Egeria densa* (Planch), and *Hydrilla verticillata* (L.F.) (Royle)) to tolerate mine waste water contaminated with multimetals concentrations, (b) to determine the metal accumulation and selectivity for each plant, and (c) to examine the plant ability to remove the toxic metal from water.

## 2. Material and Methods

### 2.1. Experimental Design and Apparatus

**Figure 1:**
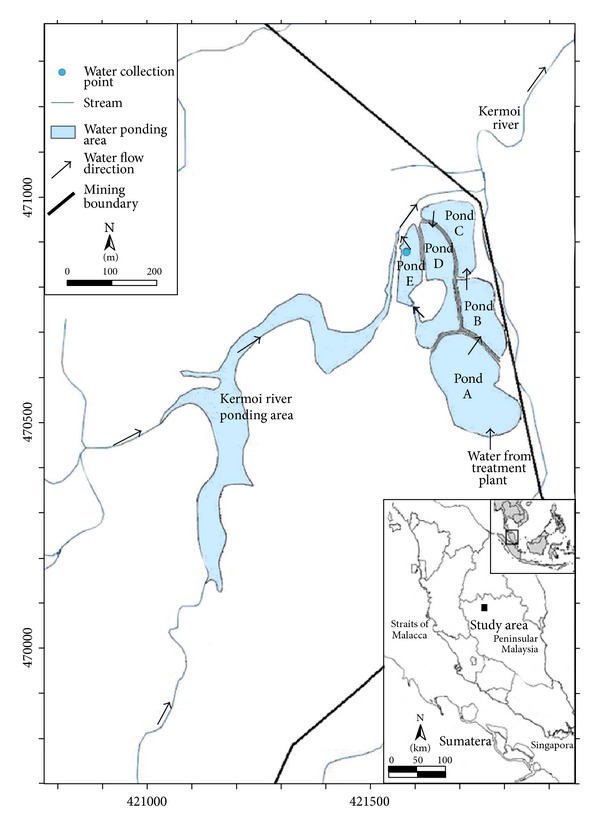
The location of the collected mine water effluent used in batch experiments.

Mine waste water effluent used in this experiment was collected from a pond in gold mining area, located at Kuala Medang area, Lipis, Pahang state, Malaysia, with latitude 4° 14′ 30′′ N to 4° 17′′ 00′′N and longitude 101° 45′ 00′′E to 101° 50′ 00′′E (Figure 1). The experimental setup consists of holding tanks, lighting systems, and aeration system. Nine plastic vessels were used in this experiment, one box for each plant. Each box (60 × 120 × 60 cm^3^) was filled with 15 L of mine water. The light intensity (625 hlx/7200 cm^2^) provided by the artificial lighting system was similar to that of natural lighting required for aquatic plants. Each lighting unit consisted of three light 40 W cool white fluorescent bulbs, 122 cm in length. The lighting system was placed on the top of each box using wooden supports in such a way that it gave a space of 30 cm clearance between the light bulbs and the water surface in the box.

The spaces between the light bulbs and the water surface are important in order to achieve a good air circulation, and further provide the heat and light required for plant growth. The plants were rinsed gently with distilled water to remove debris, and the healthy fronds were placed in plastic vessel. Aquatic macrophytes were cultured at ambient temperature under a daily regime of 12 h light for the period of 14 days.

### 2.2. Sampling and Analysis

Water samples were taken using new HDPE bottles with a 20 mL syringe from different deeps and points of the vessel for a period of 14 days. They were preserved at pH 2, following the methodology described in Section 1060 C of the Standard Methods for the Examination for Water and Wastewater [21]. The metal concentration measurement in water was carried out with Perkin Elmer ICP OES (Inductively Coupled Plasma Optical Emission Spectroscopy). A series of standard solutions (0.01, 0.25, 0.50, and 1.00 mg L^−1^) were measured as check standards. Each sample was measured (after dilution if necessary) in duplicate.

An approximately 5 g of aquatic plants was also taken simultaneously during water sampling exercise. The plants were rinsed with distilled water and dried in a convection oven for 24 h at 40°C to avoid any metal loss from plant biomass through evaporation. Plants sample were prepared by microwave digestion with an HNO_3_-H_2_O_2_ mixture in closed Teflon vessels (Multiwave 3000, Anton Paar). A 0.3 g sample was weighted on an analytical balance and placed in the digestion tube; 8.0 mL HNO_3_ (65%) and 2.0 mL H_2_O_2_ (30%) were added and placed in a microwave oven. The mixture was then kept at 15 atm pressure and 160°C for 30 min. 

The volume was brought to 50.0 mL with ultrapure water in a calibrated flask. The impurities were removed by filtration [22]. The metal elements were determined by the same analytical methods used for water samples. In each analytical batch, reagent blanks and spiked samples were included in the acid digestion to assess the accuracy of the chemical analysis. 

### 2.3. Metal Removal Efficiency and Bioconcentration Factor

The percentage of metal removal efficiency in water was calculated according to Tanhan et al. 2007 [23]:

(1) %  efficiency=C0−C1C0×100,

where *C*_0_ and *C*_1_ are initial and remaining concentrations of metal in medium (mg L^−1^).

The bioconcentration factor (BCF) was calculated as follows [24]:

(2) BCF=Metal  in  plant  biomass  (mg kg−1)  Metal  in  solution  (mg L−1).

### 2.4. Statistical Analysis

Statistical analyses of experimental data were performed using the SPSS 15.0 package for Windows. Student's *t*-tests were used to detect significant differences of metals concentrations in plant biomass and As, Al, and Zn concentrations in water samples. Evaluation of significant differences among means was performed using one-way ANOVA followed by Tukey's post-hoc test, with *P* < 0.05 indicating statistical significance. Spearman product moment correlation coefficients (*r*) were used to express the associations of quantitative variables. 

## 3. Result and Discussion

### 3.1. Physicochemical Characteristics and Metals Concentrations in Mining Water

The analysis of physicochemical properties of the mining water used in this study showed considerably high levels (Table 1) of total dissolved solids (mg L^−1^), conductivity (us/cm), and dissolved oxygen (mg L^−1^). The measured total dissolved solid was 475.3 mg L^−1^, 742.7 us/cm for conductivity and 7.77 mg L^−1^ for dissolved oxygen. The measured pH value during initial experiment was measured at 7.65 ± 0.18. As, Zn, and Al concentrations during initial experiment were measured at 0.21 ± 0.01 mg L^−1^, 0.11 ± 0.03 mg L^−1^, and 0.18 ± 0.04 mg L^−1^, respectively. The As concentration in water used in this study was higher than drinking water standard by WHO [16]. 

### 3.2. Metal Removal Efficiency

**Figure 2:**
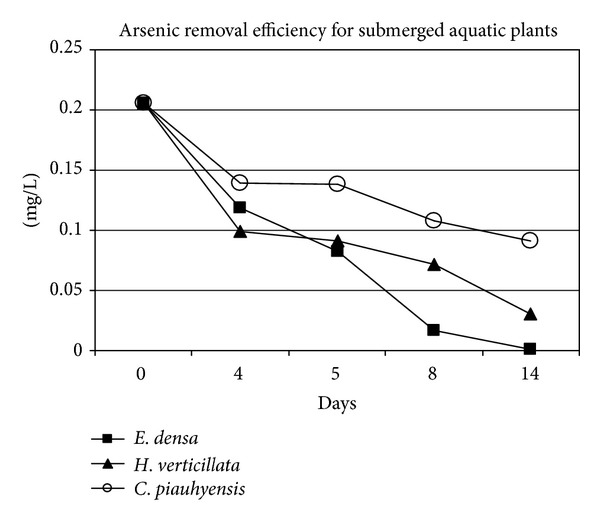
Arsenic removal trend for *C. piauhyensis*, *E. densa,* and *H. verticillata*.

**Figure 3:**
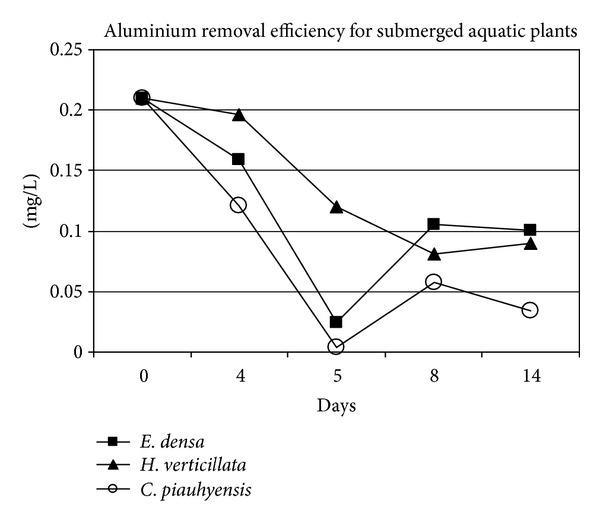
Aluminium removal trend for *C. piauhyensis*, *E. densa,* and *H. verticillata*.

**Figure 4:**
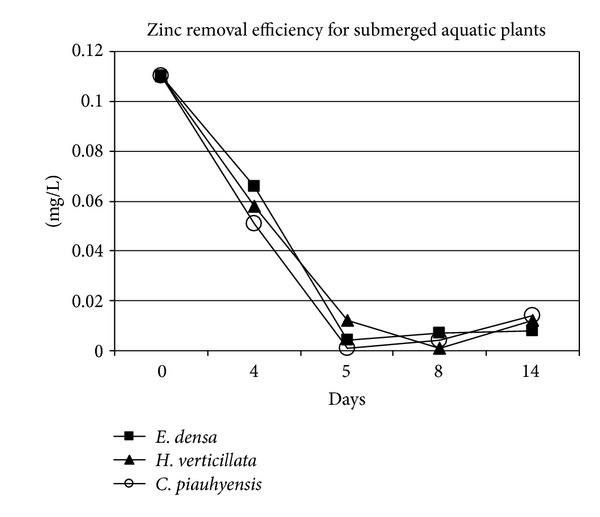
Zinc removal trend for *C. piauhyensis*, *E. densa,* and *H. verticillata*.

The results of the metal removal efficiency using mining effluent for the entire period (14 days) are shown in Figures 2, 3, and 4. After the incubation of macrophytes in the mining effluent, almost all the studied metal concentration in water decreased gradually to a minimum value for all tested plant. The highest removal efficiency (Figure 2) for As was shown by *E. densa *(95.2%) followed by *H. verticillata *(84.5%) and *C. piauhyensis *(55.8%). The considerably high As removal efficiencies by *H. verticillata* in this study can be compared with those reported by Srivastava et al. who reports significant As accumulation in plants biomass with removal of more than 70% of total As supplied after 45 days of treatments [25]. They also proposed *H. verticillata* as a promising aquatic plant for the phytoremediation of arsenic-contaminated water. Lee et al. suggest that high As concentration in *H. verticillata *is due to As uptake mechanism [26]. 

The *E. densa* also shows the highest removal efficiency for Zn (93.7%) followed by *H. verticillata* (92.3%) and *C. piauhyensis *(87.4%) (Figure 3). The highest removal efficiency for Zn by *E. densa* was also been reported by Módenes et al. [27]. Their study demonstrated that *E. densa* is able to remove more than 70% of Zn ions from solution, thus indicating the removal potential of Zn by *E. densa.* The effectiveness of *H. verticillata *in removing Zn from water has been observed by Dixit and Dhote, who studied Zn uptake and morphological changes of *H. verticillata* on a dose response basis [28]. They found that the Zn concentration in water was decreased by 60–80% after 4 weeks. The highest removal efficiency for Al (Figure 4) was demonstrated by *C. piauhyensis* (83.8%), followed by *H. verticillata* (59.1%) and *E. densa* (30.3%), respectively. 

These results show that the metal removal efficiency was varied for different aquatic plant, and *E. densa *and *H. verticillata* show a potential for As and Zn remediation, while *C. piauhyensis* demonstrates the highest Al removal effectiveness from mining waste water. Since water used in this experiment was also contaminated with many other metals and ions, the results demonstrate that the plants could maintain their potential for As, Al, and Zn accumulations even in the presence of other metals and ions, which is in conformity with observation by Srivastava et al. [29].

### 3.3. Metals Accumulation in Plants Biomass and BCF

**Figure 5:**
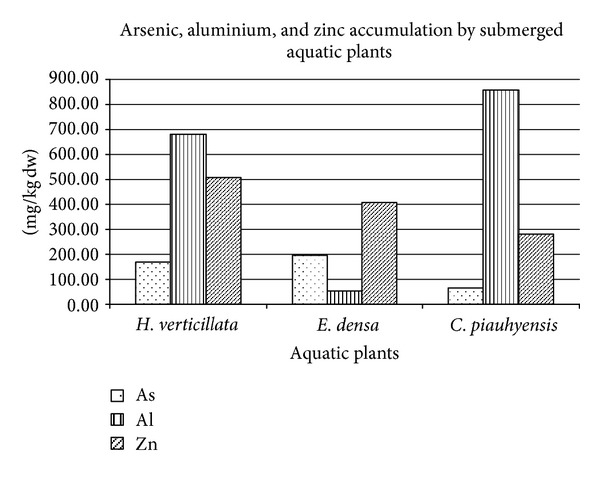
Metal accumulation (mg/kg dw) by *C. piauhyensis, E. densa*, and *H. verticillata*.

Almost all aquatic plants used in the experiment show significant accumulation of studied metal (Al, As, and Zn) during entire experimental period (Figure 5). After 14 days of mine waste water exposure, *E. densa *shows the highest As accumulation (195.95 mg kg^−1^ dw) compared to *H. verticillata* (168.75 mg kg^−1^ dw) and *C. piauhyensis *(66.86 mg kg^−1^ dw). The As accumulation in *E. densa*, however, was considerably low compared to the results of Robinson et al. who observed more than 1000 mg kg^−1^ of As accumulation in samples of *E. densa* (dry weight) collected in the Waikato river system [18]. This may be due to the extralong exposure time of *E. densa *to Waikato river water and sample, was collected from their own natural habitat. The As accumulation by *H. verticillata *in this study also can be compared to the study by Srivastava et al. who reported a slightly higher value (195 mg kg^−1^) than our findings of As accumulation in plant biomass after 15 days of exposure to As concentrated solution [29]. 

The highest Zn accumulated was demonstrated by *H. verticillata* (509.05 mg kg^−1^ dw), followed by *E. densa *(441.38 mg kg^−1^ dw) and *C. piauhyensis *(280.82 mg kg^−1^ dw). This result can be compared to the study by Srivastava et al. who demonstrated the ability of *H. verticillata *plants to accumulate significant amounts of Zn further until hyperaccumulation limit, without showing any visible symptoms of toxicity [29]. The *C. piauhyensis *has the highest Al accumulation (856.82 mg kg^−1^ dw) compared to *H. verticillata *(280.82 mg kg^−1^ dw) and* E. densa* (66.86 mg kg^−1^ dw). 

**Figure 6:**
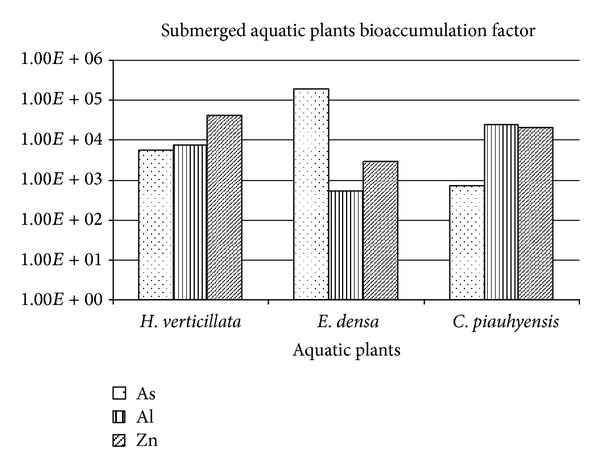
The bioaccumulation factor for *C. piauhyensis, E. densa, *and* H. verticillata* at the end of experiment (day 14th).

The bioconcentration factor (BCF) was used to quantify metal accumulation in plant biomass (Figure 6). The use of BCF is more significant compared to the metal amount accumulated in plants since it provides an index of the ability of the plants to accumulate metal element with respect to the element concentration in water [30]. At the end of experiment (14 days), the *E. densa *shows the highest As BCF value (1.96*E* + 05) compared to *H. verticillata *(5.63*E* + 03) and *C. piauhyensis *(7.35*E* + 02). The highest Zn BCF value was demonstrated by *H. verticillata *followed by E*. densa* and *C. piauhyensis *with calculated BCF value that was 4.24*E* + 04, 2.01*E* + 04, and 2.94*E* + 03, respectively. *C. piauhyensis *has the highest BCF for Al (2.52*E* + 04) compared to *E. densa *(7.55*E* + 03) and *H. verticillata *(5.40*E* + 02). The considerably high BCFs values for *E. densa* for As, *H. verticillata* for Zn, and *C. piauhyensis* for Al, reflected the ability of this plant species to accumulate these metals from mining waste water and to transport them from the roots to shoots. 

### 3.4. Relationships between As, Al, and Zn Accumulated by Aquatic Plants and Metals Removal from Mine Water

Spearman's correlations were used to analyze the relationship between the studied metal (As, Al, and Zn) accumulations by aquatic plants and metal concentration in mine water throughout experimental period. A strong significant correlation (−0.90, *P* < 0.05) was observed for As *H. verticillata* and *C. piauhyensis* (−0.92, *P* < 0.05). Although nonsignificant correlation was observed for As in *E. densa* (−0.70), the plant shows a significantly strong relationship (−0.90, *P* < 0.05) for Zn. *H. verticulata* shows a strong correlation (−0.82, nonsignificant) while a relatively low correlation was observed for Zn in *C. piauhyensis* (−0.40, nonsignificant). 

All aquatic plants show a nonsignificant low correlation for Al accumulated in plant and Al in water. A negative correlation between metal accumulated in plant and within mine water clearly demonstrated the ability of these plant in removing metal concentration in solution through adsorbing and accumulation the metal in plant mass, thus, reflecting the potential used of these plants for metal phytoremediation purposes. 

### 3.5. Plant Tolerance and Potential Plant Species for Mine Drainage Remediation

Generally, all the experimental plants did not show any reduction in the plant growth and any physical deterioration. There was no reverse effect, such as chlorosis, necrosis, and whitish-brown color, observed in the plant mass. By the end of the experiment, all the plants were survived. This shows that these plants are suitable and survived in water that contains multiconcentrations of metal, mostly found in mine drainage water. The plant survival in water with relatively high metal content is a major characteristic in selecting suitable candidate for metal phytoremediation plant that will be grown in mine drainage effluent. 

These submerged plants are also favourable to stabilize mine drainage effluent because these plants are able take up more metals, using their whole biomass as the metals uptake mechanisms can occur through entire plant surface [31]. Submerged species of aquatic macrophytes generally contained higher trace element levels than did floating-leafed species [32].

Plants with invasive behaviour such as *H. verticillata* and *E. densa* have a wide ecological range. These plants possess a more promising result as a candidate for potential phytoremediation. This is due to their nature that easy to grown and only need minimal treatment. At a nutrient rich situation, these plants, however, cannot been planted together because of *H. verticillata* is a better competitor than *E. densa* and will out-compete successfully *E. densa* [33]. These plants were also suitable for remediation of water with elevated As concentration as proposed by Srivastava et al. [25] using *H. verticillata* and *E. densa* by Robinson et al. [18]. 

## 4. Conclusion

Results of this study indicate that *E. densa* and *H. verticillata* have relatively high As and Zn accumulation in plant mass and are able to remove these metal from mine water effluent. On the other hand, *C. piauhyensis *demonstrates the highest Al removed from mine water and accumulated in plant mass. The present study also provides an insight into the mechanisms of As, Zn, and Al accumulation and resistance in *E. densa*, *H. verticillata,* and *C. piauhyensis, *respectively. The ability of these plants to accumulate the studied metals and survive throughout the experiment demonstrates the potential of these plant to remediate metal enriched water especially for mine drainage effluent treatments. 

## Figures and Tables

**Table 1:** Physicochemical characteristic for mine waste water.

Parameters	Value	WHO, 2008 [16] (mg L^−1^)
pH	7.65 ± 0.18	
TDS (mg L^−1^)	475.3 ± 1.29	
Conductivity (us/cm)	742.7 ± 5.95	
DO (mg L^−1^)	7.77 ± 0.03	
K (mg L^−1^)	7.38 ± 0.24	
Ca (mg L^−1^)	34.05 ± 0.69	
Mg (mg L^−1^)	2.81 ± 0.08	
Na (mg L^−1^)	21.43 ± 1.26	
Al (mg L^−1^)	0.21 ± 0.04	0.2
Zn (mg L^−1^)	0.11 ± 0.03	3
As (mg L^−1^)	0.21 ± 0.01	0.01
